# The Rhizosphere Microbiome: A Key Mediator of Crop Responses to Fertilization Strategies

**DOI:** 10.3390/plants15172711

**Published:** 2026-09-03

**Authors:** Zhihui Zhao, Qihua Wu, Zerong Sun, Wenling Zhou, Junhua Ao

**Affiliations:** 1 International Magnesium Institute, College of Resources and Environment, Fujian Agriculture and Forestry University, Fuzhou 510220, China; 2 Institute of Nanfan & Seed Industry, Guangdong Academy of Sciences, Guangzhou 510316, China

**Keywords:** rhizosphere microbiome, fertilization strategies microbial community assembly, root exudates, plant growth-promoting rhizobacteria (*PGPR*), sustainable agriculture

## Abstract

The rhizosphere microbiome, the plant’s “second genome” is pivotal for crop nutrient acquisition, health, and stress responses. While fertilization ensures high agricultural yields, a key challenge is reshaping this microbiome to boost crop performance. This review synthesizes how mineral, organic, and bio-organic/microbial inoculant fertilizers affect rhizosphere microbial structure, diversity, and function. Long-term excessive mineral fertilizers (especially nitrogen) reduce microbial diversity, diminish beneficial groups (e.g., diazotrophs, *PGPR*), and disrupt microbial networks via soil acidification and altered root exudates, causing continuous cropping obstacles. In contrast, organic fertilizers improve soil microenvironments, maintaining high microbial diversity, enriching beneficial taxa (e.g., *Proteobacteria, Actinobacteria*), and enhancing community complexity. Bio-organic fertilizers/microbial inoculants “engineer” the microbiome by introducing exogenous beneficial microbes (e.g., *Bacillus, Pseudomonas, AMF*), directly promoting growth, suppressing diseases, and “reconditioning” indigenous beneficial communities. We also clarify how fertilization regulates plant-microbe dialog via root exudates and rhizosphere chemistry (e.g., pH, ion balance), discuss current challenges (causality, lab-to-field translation, genotype-microbiome-fertilization interactions), and outline future directions. Integrating rhizosphere microbiome management into fertilization is crucial for reducing chemical fertilizer reliance and advancing agricultural green transformation.

## 1. Introduction

The rhizosphere, the narrow soil zone intensely influenced by root activities, is a highly dynamic interface for plant–soil microbe interactions. It serves not only as a hotspot for nutrient acquisition but also hosts the rhizosphere microbiome, often regarded as the plant’s “second genome” whose complex microbial communities are crucial for plant health [1,2]. These microbial assemblages constitute the ‘first line of defense’ for plants against soil-borne pathogens, protecting their hosts through various mechanisms, including competition, antagonism, and induced systemic resistance [3]. Consequently, a deep understanding of the rhizosphere micro-ecosystem is fundamental to unlocking plant productivity potential and achieving agricultural sustainability [2].

**Figure 1:**
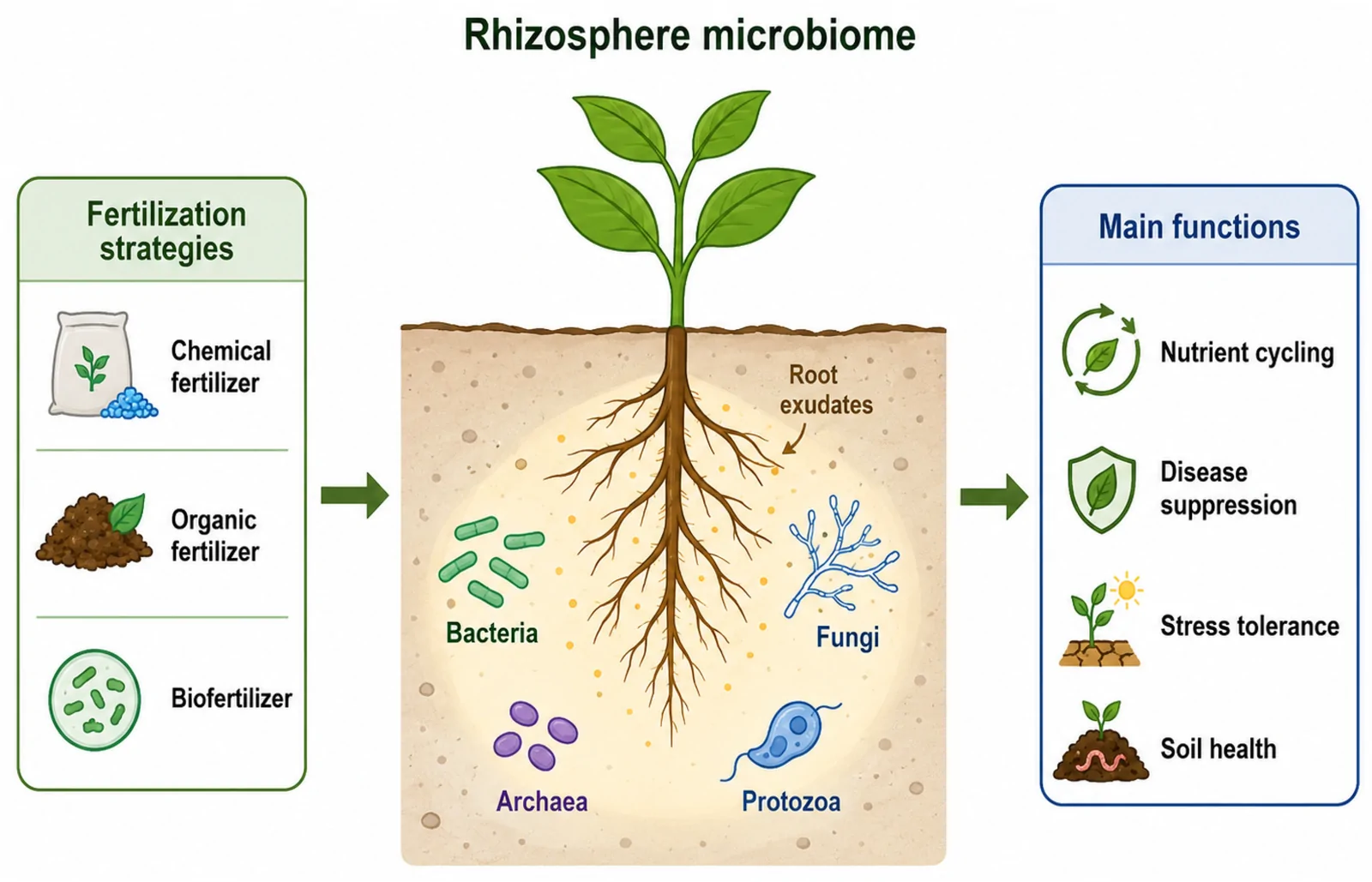
Schematic Diagram of Fertilization-Regulated Rhizosphere Microbiome and Its Core Functions.

The rhizosphere microbiome exhibits astonishing diversity, encompassing tens of thousands of species of bacteria, fungi, archaea, and protozoa [1,4]. These microbes are not randomly assembled but form specific communities shaped by precise plant genotypic regulation [5]. Functioning as ‘microbial engines’ of the ecosystem, they drive a series of core processes: for instance, bacteria and archaea are involved in nitrogen fixation, nitrification/denitrification, while phosphate- and potassium-solubilizing microbes activate insoluble soil nutrients, and arbuscular mycorrhizal fungi (*AMF*) significantly expand the root absorption range [6,7]. Furthermore, the rhizosphere microbiome aids plants in resisting biotic (e.g., *pathogen infection*) and abiotic stresses (e.g., drought, heavy metal contamination) and contributes to soil remediation by degrading organic pollutants [8,9] (Figure 1).

Fertilization is one of the most crucial management practices in modern agriculture, aimed at supplementing soil nutrients and ensuring high crop yields [10]. However, long-term excessive application of chemical fertilizers, especially nitrogen, while potentially boosting short-term yield, can lead to negative consequences such as soil acidification, compaction, organic matter decline, and microbial community structure dysbiosis [11,12]. Such deterioration of soil health directly impacts the rhizosphere micro-ecosystem [13]. Research indicates that different fertilization strategies (e.g., chemical fertilizers, organic fertilizers, microbial fertilizers) significantly alter the community structure and functional gene expression of rhizosphere microbes [14,15]. For example, organic fertilizer application tends to increase microbial diversity and the abundance of beneficial microbes (e.g., *Proteobacteria, Actinobacteria*), whereas sole reliance on chemical fertilizers may promote oligotrophic taxa and suppress key nutrient cycling functions [12,16]. Therefore, investigating how fertilization specifically influences this critical interface is essential for optimizing fertilization strategies [17].

Despite significant advances in soil microbiome research, current studies often focus on macro-descriptions of bulk soil microbial communities, lacking systematic compilation and comparison of the specific responses of the rhizosphere—this distinct micro-domain—to fertilization management [5,18]. In particular, the intrinsic mechanisms by which different fertilization regimes (e.g., combined organic-inorganic application, introduction of microbial fertilizers) precisely affect the functional networks of the rhizosphere microbiome (such as carbon, nitrogen, and phosphorus cycling, and pathogen suppression) via regulating signaling molecules like root exudates remain poorly understood [19,20]. This knowledge gap limits our ability to precisely manage the rhizosphere microbiome for enhanced fertilizer use efficiency and crop stress resilience.

This review aims to systematically summarize and critically analyze recent research progress on how different fertilization strategies influence the structure and function of the plant rhizosphere microbiome. It will elaborate on the composition and core functions of the rhizosphere microbiome, with a focus on how chemical, organic, and biological fertilizers reshape these microbial communities and the underlying regulatory mechanisms. Finally, the challenges associated with manipulating the rhizosphere microbiome through fertilization strategies and the application prospects for promoting sustainable agricultural development will be discussed. By integrating multi-omics technologies and ecological theory, this review seeks to provide a theoretical basis and new perspectives for achieving fertilizer reduction and efficiency gain, along with healthy crop cultivation, via rhizosphere microbiome management.

## 2. Structure and Function of the Rhizosphere Microbial Community

### 2.1. Structural Characteristics of the Rhizosphere Microbial Community

The structure of the rhizosphere microbial community is not randomly assembled but is a result of strict filtering by plant, soil, and environmental factors, presenting unique diversity patterns, a core microbiome, and distinct community assembly processes [21,22,23].

The rhizosphere microbiome possesses astonishing diversity, encompassing tens of thousands of species [2]. However, compared to the surrounding bulk soil, microbial diversity in the rhizosphere is typically lower. This discrepancy stems from the selective screening of microorganisms by plant roots from the soil microbial “seed bank,” leading to the enrichment of specific microbial taxa in the rhizosphere [24]. This selective pressure results in a community assembly process dominated by stochastic processes, forming highly modular yet unstable bacterial networks, reflecting microbial adaptation to and interactions within the dynamic rhizosphere conditions [24]. Metagenomic studies further reveal that the assembly of the rhizosphere microbiome is a two-step selection process: root deposits first drive initial, substrate-based community changes in the rhizosphere, which then combine with host genotype-dependent fine-tuning to ultimately select for a specific endophytic microbial combination within the roots [25].

Under this selective pressure, plants form a relatively stable core microbiome. Research indicates that different plant species harbor distinct microbial assemblages even when grown in the same soil [1,2]. Strategies like serial passage allow for the derivation of simplified yet representative synthetic communities from the complex root microbiome [26]. For instance, a synthetic community obtained from the wheat root microbiome, comprising a limited number of bacterial strains, not only successfully colonized the roots but also demonstrated an ability to suppress pathogenic fungi, with *Enterobacter* identified as a keystone species crucial for maintaining community stability [26]. Members of this core microbiome, such as Proteobacteria, Actinobacteria, Bacteroidetes, and Firmicutes, play key roles in nutrient cycling and plant health [25].

Plants can also actively “recruit” beneficial microorganisms through signaling molecules like root exudates. For example, seed-borne microorganisms can surpass native soil microbes to become a dominant source in root community assembly. The genomes of these seed-originating microbes are enriched with host-associated functional traits, such as the ability to degrade key sugars for niche differentiation and cross-feeding interactions that support partner strains, thus playing a significant role in microbial succession and community assembly [27].

### 2.2. Core Functions of the Rhizosphere Microbiome

The rhizosphere microbial community directly or indirectly promotes plant growth, enhances stress resistance, and improves the rhizosphere microenvironment through a suite of core functions [28,29,30].

Nutrient solubilization and cycling represent one of the most fundamental functions of the rhizosphere microbiome. Microorganisms act as “microbial engines” of soil nutrient cycling, driving the transformation of key elements such as carbon, nitrogen, and phosphorus [31]. Studies have shown that the abundance of microbial functional genes is closely correlated with the nutrient cycling indicators they proxy [32]. For instance, long-term fertilization significantly alters the functional profiles of soil carbon and nitrogen cycling. The application of organic fertilizer enriches genes associated with carbon fixation and assimilatory/dissimilatory nitrate reduction, while chemical fertilizer promotes the enrichment of genes related to nitrogen degradation, nitrification, and anammox [11]. The functional potential of the rhizosphere microbiome, particularly of taxa involved in carbon degradation, nitrification, and organic phosphorus mineralization, shows a significant positive correlation with ecosystem multifunctionality [33].

In terms of phytohormone regulation, rhizosphere microbes directly influence plant growth and development by producing or modulating plant hormones, such as auxin. Research has demonstrated that inoculating with plant growth-promoting rhizobacteria (PGPR) can enhance root activity and promote crop nutrient uptake [34]. Synthetic microbial communities (SynComs), particularly those composed of moderately related strains, exhibit superior plant growth-promoting effects by enhancing plant growth, root colonization, indole-3-acetic acid (IAA) production, and siderophore production [35].

Induced systemic resistance (ISR) is a key mechanism through which the rhizosphere microbiome enhances plant health. Rhizosphere microbes can help plants resist pathogen invasion by inducing systemic resistance [1,24]. Core microbial species influence plant health by enhancing cooperation and network complexity within the soil microbial community [36]. For example, in continuous watermelon cropping systems, the introduction of more healthy soil can reduce the plant disease index by increasing the stability and complexity of the microbial network. The positive cohesion, which reflects the degree of cooperation among microbes, is negatively correlated with the plant disease index [36].

Finally, the rhizosphere microbiome participates in modulating rhizosphere structure. Roots and the associated rhizosphere drive the formation of soil structure from the rhizosphere to the detritusphere by shaping soil physical architecture [37]. Root growth, the production of root exudates and root hairs, and soil microbial activity collectively regulate this process. Root-secreted extracellular polymeric substances (EPS) contribute to aggregate formation, while microbes further stabilize soil structure through their hyphae and metabolic products, thereby improving rhizosphere aeration and water-holding capacity to create a more favorable growth environment for plants [37] (Table 1).

## 3. Effects of Different Fertilization Strategies on the Rhizosphere Microbiome

### 3.1. Impact of Mineral Fertilizers

The application of mineral fertilizers, particularly long-term and excessive use, exerts profound and complex influences on the rhizosphere microbial community by altering the physicochemical properties of the rhizosphere soil (e.g., pH, electrical conductivity, nutrient availability) and the physiological status of the plant. These effects are primarily manifested in aspects such as microbial community structure, diversity, specific functional groups, and microbial interaction networks.

Long-term application of mineral fertilizers, especially nitrogen fertilizers, is a key driver of changes in rhizosphere microbial community structure. Research indicates that the application of inorganic nitrogen fertilizer significantly altered the taxonomic and predicted functional structure of the wheat rhizosphere microbiome [38]. Excessive nitrogen application (N300, 300 kg N ha^−2^) significantly decreased the *nifH* gene copy number, species richness and Shannon index of diazotrophs in black soil maize rhizosphere by 53.7–79.7%, 37.2–47.6% and 20.0–31.6%, respectively, and altered diazotroph community composition with reduced relative abundance of *Burkholderia* but elevated abundance of *Sphingobium* [39]. This shift in community structure was closely correlated with decreased soil pH and increased electrical conductivity [39]. A 25-year fertilization trial in acidic red soil also found that the application of nitrogen fertilizer alone or in combination with phosphorus and potassium (NPK) reduced both maize biomass and the *nifH* gene copy number of rhizosphere diazotrophs, whereas the application of only phosphorus and potassium (PK) increased them, indicating a suppressive effect of nitrogen input on the diazotrophic community [40].

The impact of mineral fertilizers on diversity presents a complex picture. An 8-year long-term nitrogen gradient experiment on wheat showed that the alpha diversity of rhizosphere bacteria increased with the historical nitrogen input rate but stabilized after the rate exceeded 280 kg N ha^−1^ year^−1^ [41]. More studies report negative effects of mineral fertilizers, particularly inorganic nitrogen, on microbial diversity. Long-term application of inorganic fertilizers led to soil acidification in the walnut rhizosphere, reducing fungal diversity [42]. Studies on the wheat rhizosphere also found that inorganic nitrogen fertilizer application reduced bacterial richness and diversity, while manure treatment maintained higher diversity [38]. Excessive nitrogen fertilizer imposes greater environmental filtering pressure on the diazotroph community by lowering soil pH and increasing salinity, resulting in a higher degree of phylogenetic clustering (indicating stronger environmental selection) [39,41].

The impact of mineral fertilizers on microbial function is particularly significant. Quantitative microbiome analysis revealed that unbalanced mineral fertilizer treatments (lacking N, P, or K) significantly affected the assembly of soybean rhizosphere bacteria. The nitrogen-deficient treatment caused the bacterial community to diverge from other fertilized treatments, while phosphorus deficiency hindered the total load and turnover of rhizosphere bacteria [43]. Mineral fertilizer application alters the profile of microbial functional genes. For example, the nitrogen application rate influenced the relative and total abundance of nitrogen-cycling-related genes (e.g., nitrification genes *pmoA-amoA* and denitrification genes *nirK*, *nosZ*) in the maize rhizosphere [44]. A high nitrogen application rate increased the total amount of root exudates and raised the overall abundance of rhizosphere bacteria, which might be one reason for the decreased fertilizer use efficiency and increased nitrogen loss under high nitrogen levels [44]. Furthermore, culture-dependent techniques confirmed that compared to no fertilization, the application of inorganic fertilizers significantly reduced the proportion of rhizobacteria isolated from the wheat rhizosphere that exhibited plant growth-promoting (PGP) traits such as phosphate solubilization and potassium release [45].

From the perspective of microbial interactions, long-term mineral fertilization affects community stability and complexity. Studies have found that soils with a history of high nitrogen input subject their rhizosphere bacterial communities to stronger environmental filtering pressure, leading to a higher degree of phylogenetic clustering [41]. Consecutive application of mineral fertilizers alters the co-occurrence network structure of bacterial-fungal communities. For instance, in a continuous watermelon cropping system, the persistent use of mineral fertilizers suppressed the abundance of key ecological clusters containing beneficial bacteria (e.g., *Lysobacter* and *Haliangium*) within the bacterial co-occurrence network, thereby exacerbating the obstacles associated with continuous monocropping [46]. In contrast, reduced mineral fertilizer application combined with organic amendments (e.g., straw, organic manure) can improve soil fertility, increase the abundance of bacteria and fungi, alter the community structure, and lead to more complex and stable microbial networks [47].

In addition to nitrogen, phosphorus (P) and potassium (K) fertilizers also significantly influence the rhizosphere microbial community [48]. Long-term P deficiency sup-presses microbial metabolic activity and reduces the abundance of phosphatase-producing microbes (e.g., *Burkholderia* and *Pseudomonas*), thereby affecting soil P availability [49]. Studies have shown that under P deficiency, plants increase the secretion of organic acids to recruit phosphate-solubilizing bacteria (PSB), partially alleviating P stress [50]. K deficiency affects microbial osmoregulation and reduces the colonization ability of certain PGPR strains. Moreover, long-term unbalanced fertilization (e.g., NPK lacking P or K) simplifies microbial network structures and reduces functional gene abundance, ultimately impairing crop nutrient uptake [51]. Therefore, systematic studies on the roles of P and K fertilizers in regulating the rhizosphere microbiome are urgently needed [52].

In summary, the influence of mineral fertilizers, particularly nitrogen fertilizers, on the rhizosphere microbiome is characterized by: reducing soil pH, thereby creating environmental stress for the microbial community; suppressing the abundance and diversity of beneficial functional groups such as diazotrophs; altering the functional gene profiles associated with microbially mediated nutrient cycling and disrupting the stability of microbial interaction networks. These effects subsequently constrain the maintenance of soil health and the sustainability of agricultural production.

### 3.2. Impact of Organic Fertilizers

In contrast to mineral fertilizers, organic fertilizers (e.g., farmyard manure, green manure, compost) introduce abundant organic matter and diverse nutrients into the soil ecosystem, thereby exerting distinctly different effects on the rhizosphere microbial community [53,54]. Their mechanisms of action primarily involve improving soil physicochemical properties, providing diversified energy sources, and modulating the microbial growth environment [55,56].

Long-term application of organic fertilizers can significantly improve soil physicochemical properties, creating a more favorable habitat for microorganisms. A 10-year maize-green manure intercropping trial in the Hexi Corridor demonstrated that green manure application significantly increased soil pH, nutrient content, and enzyme activities, while simultaneously reducing the relative abundance of potential plant pathogens. These improvements were closely associated with changes in microbial community structure and activity [57]. A study on ameliorating acidified soil also found that the application of organic fertilizer (e.g., farmyard manure) combined with lime increased soil pH and significantly enriched the abundance of beneficial microorganisms such as *Actinobacteria* and *Proteobacteria* in the rhizosphere [58]. These optimizations of soil properties lay the foundation for enhanced microbial diversity and the proliferation of beneficial microbes.

One of the most notable impacts of organic fertilizers is the enhancement of rhizosphere microbial diversity. Long-term stationary experiments have shown that continuous application of organic fertilizer maintains higher bacterial and fungal diversity compared to mineral fertilizer alone in walnut orchards [59]. Short-term application of organic fertilizer also significantly increased the Chao1 and Shannon indices of rhizosphere bacteria in tea garden soils [60]. This enhancement in diversity stems from the complex and recalcitrant organic carbon sources provided by organic fertilizers, which create diverse ecological niches, supporting the coexistence and development of a wider variety of microorganisms and thereby buffering the community stability against environmental fluctuations [59,60].

Organic fertilizers show promise in regulating specific functional microbial groups. A 31-year rice-rice-green manure rotation system study found that green manure rotation significantly altered the structure of the rice rhizosphere microbial community and enriched known plant growth-promoting bacteria, such as *Acinetobacter* and *Pseudomonas* [61]. In a peanut cropping system, long-term organic amendment changed the microbial co-occurrence patterns related to phosphorus cycling, enriching key OTUs (Operational Taxonomic Units) with potential for synergistic C-P and N-P transformations (e.g., *Chitinophaga pinensis* and *Nitrospira moscoviensis*), thereby promoting rhizosphere phosphorus cycling [62]. Metagenomic studies further revealed that compared to mineral fertilizer, organic fertilizer treatment enriched more functional genes involved in nitrogen cycling (e.g., denitrification) and carbon degradation (particularly recalcitrant carbon) [63].

Organic fertilizers can also significantly alter inter-microbial interactions, enhancing community complexity and stability. Network analysis indicates that long-term organic fertilization increases the size and connectivity of bacterial co-occurrence networks [63]. Research in kiwifruit orchards found that organic fertilizer application stimulated the formation of functional microbial groups and activated antagonistic or mutualistic relationships among microbial taxa, thereby enhancing the complexity of the microbial network [64]. This complex network structure is often positively correlated with the enhancement of soil ecosystem functions and crop yield increases [64,65].

Furthermore, organic fertilizers play a role in inducing systemic resistance. Although they introduce fewer direct probiotics compared to bio-organic fertilizers, they indirectly enhance plant health by reshaping the soil microbial community. Studies have shown that organic amendment can indirectly promote the activity of enzymes related to root disease resistance (e.g., phenylalanine ammonia-lyase and superoxide dismutase) by improving soil chemical properties and the microbial community structure [58]. Under drought stress, the soil microbial community in organic fertilizer treatments exhibited higher resistance and resilience. Its bacterial diversity was higher than that in mineral fertilizer treatments during the drought period and showed higher ecological recovery after rewatering, which helps suppress pathogen-related functions [66].

Through their “soil conditioning” function, organic fertilizers positively influence the rhizosphere microbiome in multiple ways: they improve the soil microenvironment, providing a substrate for microbial proliferation; supply diverse carbon sources, supporting higher microbial diversity; selectively enrich beneficial microbial taxa with specific nutrient cycling functions; and promote the formation of more complex and stable interaction networks among microbes [67,68]. Ultimately, this enhances the buffer capacity and service functions of the soil ecosystem, creating a healthy rhizosphere environment for crop growth [69].

### 3.3. Impact of Bio-Organic Fertilizers and Microbial Inoculants

Bio-organic fertilizers (BOFs) and microbial inoculants represent an advanced form of fertilization strategy. They not only supply organic nutrients but also directly introduce exogenous beneficial microorganisms (e.g., plant growth-promoting rhizobacteria—PGPR, arbuscular mycorrhizal fungi—AMF), aiming to proactively design and manipulate the rhizosphere microbiome for a more precise enhancement of soil ecological functions and services [70,71]. The core mechanism of their impact lies in the interaction between the introduced microorganisms and the indigenous microbial community, as well as the targeted regulation of the overall rhizosphere microenvironment [72,73].

The most direct effect of BOFs and microbial inoculants is their successful colonization and subsequent influence on the structure of the rhizosphere microbial community. In a study on suppressing banana Fusarium wilt, the application of BOF containing *Bacillus amyloliquefaciens* NJN-6 not only enabled the successful colonization and stable abundance of the strain in the rhizosphere but also significantly increased bacterial richness and diversity, while reducing fungal abundance. It enriched beneficial taxa such as *Sphingobium*, *Dyadobacter*, and *Cryptococcus*, and decreased the relative abundance of pathogens like *Fusarium oxysporum* [74]. In a peanut continuous cropping system, the application of microbial agents (MA) and microbial fertilizers (MF) increased the richness of rhizosphere soil bacteria and altered the community structure by increasing the abundance of potentially beneficial bacteria (e.g., *Bradyrhizobium*, *Rhizobium*, *Burkholderia*) and fungi (e.g., *Trichoderma*), while reducing the abundance of pathogenic fungi (e.g., *Penicillium*, *Fusarium*) [14]. The success of colonization and the magnitude of the effect are closely related to the compatibility between the introduced strain and the indigenous microbial flora. Research has shown that a synthetic community (SynCom) composed of 21 bacterial strains screened from local soil exhibited a stronger “home-field advantage” in low-fertility soil compared to commercial PGPR inoculants. The superior growth-promoting effect on maize was associated with the SynCom’s stronger colonization ability in the rhizosphere and more positive interactions with the indigenous community [75].

The introduction of these exogenous microorganisms often functions indirectly by modulating the indigenous microbial community, rather than relying solely on their direct effects. Studies on banana Fusarium wilt have revealed the disease suppression mechanism of BOFs: the introduced probiotics (e.g., *Bacillus* and *Trichoderma*) primarily induce systemic resistance by altering the structure and function of the soil microbiome, rather than directly inhibiting pathogens [74,76]. Specifically, the application of BOFs enriches indigenous microbial taxa with the ability to produce secondary metabolites, such as members of the *Sphingomonadaceae* and *Xanthomonadaceae* families. These taxa are activated upon pathogen invasion, thereby suppressing *Ralstonia solanacearum* [77]. Similarly, BOF containing *Trichoderma asperellum* NJAU4742 can stimulate beneficial indigenous microbial populations in the soil, such as *Pseudomonas*, to suppress diseases through synergistic interactions among microorganisms [78]. This mechanism of “priming” or “reconditioning” the indigenous community is considered key to the long-term and stable effects of BOFs [76,77].

By introducing specific functional strains, BOFs and inoculants can significantly enhance particular functions of the rhizosphere microbiome. For instance, BOFs containing *Bacillus velezensis* SQR9 or *Trichoderma harzianum* NJAU4742 tend to increase the levels of soil organic matter, available phosphorus, nitrogen, potassium, iron, zinc, and other nutrients by enriching functional genera such as *Urebacillus* and *Streptomyces* [79]. In the remediation of multi-metal contaminated saline-alkali soil, the synergistic application of AMF and BOF significantly enriched key taxa with potential for pollutant degradation and nutrient cycling, such as *Chloroflexi* and *Patescibacteria* [80]. Inoculation with *Pseudomonas plecoglossicida* induced the root secretion of amino acids and carbohydrates, recruiting beneficial bacteria like *Pseudomonas* and *Sphingomonas*, which collectively enhanced the degradation of BDE-47 in the rhizosphere [81]. Under saline-alkali stress, the microbial inoculant GB03 increased the abundance of beneficial bacteria such as *Acidobacteriota*, *Bdellovibrionota*, and *Bacillus*, thereby alleviating stress and improving grape fruit quality [82].

The application of BOFs and inoculants can also significantly alter microbial interaction networks, enhancing their complexity and stability. Long-term application of BOF containing *Trichoderma* increased the complexity and connectivity of the bacterial co-occurrence network in the kiwifruit rhizosphere, forming a more functional microbiota [64]. In the pear rhizosphere, BOF containing *Bacillus velezensis* SQR9 optimized the microbial co-occurrence network and enhanced metabolic functions related to carbon/nitrogen fixation, phosphorus solubilization, and nitrification. This more complex network was directly correlated with improved soil nutrient availability [83]. Inoculation with AMF also enhanced the complexity and stability of the maize rhizosphere microbial ecological network under drought stress, indicating tighter interactions among microorganisms [84]. It is noteworthy that composite inoculation (e.g., combined AMF and PGPR) often produces synergistic effects superior to single inoculation. For example, co-inoculation with AMF and *Pseudomonas* sp. SG29 showed the best promoting effect on tobacco seedling growth, resulting in the highest rhizosphere microbial diversity and significantly promoting the colonization of both AMF and *Pseudomonas* [85]. Similarly, the synergistic inoculation of AMF and PGPR significantly enhanced alfalfa’s tolerance to saline-alkali stress by strengthening the link between rhizosphere metabolites and the microbiome [86].

Through “engineering” strategies, bio-organic fertilizers and microbial inoculants go beyond the soil amendment role of conventional organic fertilizers. Their core impact pathways include: the successful colonization of exogenous functional microorganisms, making them new members of the rhizosphere microbiome; regulation of the structure and function of the indigenous microbial community through “priming” or “reconditioning” mechanisms; direct or indirect enhancement of specific microbe-driven soil functions (e.g., nutrient cycling, pollution remediation, stress resistance); and optimization of microbial interaction networks, improving the functional redundancy and stability of the entire microbial community. These mechanisms work together, providing powerful tools for the targeted manipulation and sustainable production of agricultural ecosystems.

### 3.4. Comprehensive Comparison of Fertilization Strategies

Fertilization strategies exert profound influences on the rhizosphere microbial community, as different fertilization practices directly regulate microbial diversity, structure, and function by altering the soil environment. Long-term application of mineral fertilizers can lead to soil acidification and reduced microbial diversity; for instance, it decreases the abundance of diazotrophs, thereby potentially inhibiting plant growth [38]. In contrast, organic fertilizers, such as manure, can significantly enhance the abundance and enzyme activities of rhizosphere microorganisms, promote nutrient cycling, and enrich beneficial microbes like *Proteobacteria* [87]. Green manure intercropping further improves soil physicochemical properties, enhances the complexity of microbial networks, and increases plant stress resistance [57]. Biofertilizers, by introducing specific probiotics (e.g., *Bacillus* spp.), directly reshape the rhizosphere microbial community and enhance disease suppression capacity [14]. These distinctions underscore the critical role of sustainable fertilization strategies in optimizing the rhizosphere micro-ecology (Table 2).

## 4. Mechanisms of Fertilization in Regulating the Rhizosphere Microbiome and Crop Effects

### 4.1. Mechanisms of Regulating the Rhizosphere Dialog

Fertilization strategies directly regulate the “rhizosphere dialog” between plants and microorganisms by altering the composition of root exudates and the chemical environment of the rhizosphere, thereby driving the restructuring of the microbial community [96]. Root exudates serve as key communication media between plants and microbes, and their composition and quantity are significantly influenced by fertilization practices. For instance, long-term application of chemical nitrogen fertilizers (e.g., nitrate) can induce cucumber roots to increase the release of pathogen-preferential exudates such as gluconic acid and sorbitol [97,98]. In contrast, an adequate nitrate supply promotes the secretion of antibacterial compounds like guanidinosuccinic acid and behenic acid, thereby selectively enriching beneficial bacteria and inhibiting pathogen growth [99]. Conversely, treatments with organic and bio-organic fertilizers can increase the concentrations of sugars, organic acids, and secondary metabolites (e.g., flavonoids) in crop root exudates. These compounds act as specific chemotactic signals that attract plant growth-promoting rhizobacteria (PGPR), such as *Pseudomonas* and *Bacillus*, ultimately optimizing microbial community function [100]. In citrus orchards, the application of green manures like rice straw increases the content of organic acids and flavonoids in root exudates. These substances significantly enhance nitrogen cycling efficiency by modulating the rhizosphere C/N ratio and the expression of microbial nitrogen fixation genes (e.g., *nifH*) [101].

Fertilization also directly filters microbial communities by altering rhizosphere pH and ion balance. The long-term use of chemical fertilizers often leads to soil acidification, lowering rhizosphere pH. This suppresses the activity of beneficial microorganisms such as *Actinobacteria* while promoting the proliferation of acid-tolerant phyla like *Acidobacteria* [102]. In contrast, the addition of organic fertilizers and biochar can effectively neutralize soil acidity, raise rhizosphere pH, and provide abundant organic carbon sources, thereby enriching bacterial phyla with nutrient-mobilizing functions, such as *Proteobacteria* and *Firmicutes* [103]. In saline-alkali soils, the application of bio-organic fertilizers containing *Paenibacillus mucilaginosus* can lower rhizosphere pH through bacterial secretion of organic acids, enhancing the solubility of nutrients like iron and phosphorus. Simultaneously, it helps regulate ion balance, mitigating the inhibitory effects of salt stress on microorganisms [104]. These changes in chemical factors collectively create a rhizosphere microenvironment that is favorable for the colonization of plant-beneficial microbial communities.

### 4.2. Feedback Effects on Crop Growth

Fertilization strategies, through their regulation of the rhizosphere microbiome, exert significant positive or negative feedback effects on crop growth. In terms of positive feedback, optimized fertilization can enhance nutrient use efficiency and subsequently increase crop yield by reshaping the microbial community [102,105,106]. For instance, in a peanut-rice rotation system, the application of a compound microbial inoculant containing nitrogen-fixing rhizobia (e.g., *Pararhizobium giardinii*) and endophytes (e.g., *Serratia plymuthica*) significantly increased nodule nitrogenase activity (evidenced by a 200% increase in *nifH* gene expression), promoted plant uptake of nitrogen and phosphorus, and resulted in a peanut grain yield of 1168 kg ha^−1^, which was significantly higher than that achieved with chemical fertilizer alone [107]. In cucumber continuous cropping soil, the application of bio-organic fertilizer amended with *Trichoderma* enriched beneficial bacteria (e.g., *Sphingomonas*), enhanced the activities of soil phosphatase and urease, consequently improved phosphorus use efficiency, increased cucumber biomass by 20.39%, and reduced the incidence of Fusarium wilt [67]. These cases demonstrate that fertilization strategies can directly promote crop nutrient acquisition and disease resistance by regulating microbial community functions.

Inappropriate fertilization practices may trigger negative feedback, leading to dysfunction of the microbial community, thereby exacerbating continuous cropping obstacles or disease outbreaks. Long-term excessive application of chemical nitrogen fertilizer can cause soil acidification, suppress the abundance of beneficial microorganisms such as arbuscular mycorrhizal fungi (AMF) and diazotrophs, and reduce the complexity of the soil microbial network, ultimately diminishing crop nutrient acquisition and aggravating continuous cropping obstacles [108]. In a continuous tomato cropping system, the sole application of chemical fertilizer reduced rhizosphere microbial diversity, decreased the abundance of antagonistic bacteria like *Pseudomonas*, and increased the relative abundance of pathogens such as *Fusarium*, finally intensifying the occurrence of bacterial wilt [109]. Furthermore, imbalanced fertilization can disrupt the interactive networks within the rhizosphere microbiome, decrease microbial functional redundancy, weaken the soil’s buffering capacity against biotic stresses, and consequently lead to crop yield reduction and quality deterioration [110]. Therefore, scientific fertilization strategies must consider the stability of microbial community functions to maximize the sustainability of crop production (Table 3).

## 5. Challenges and Future Perspectives

### 5.1. Challenges in Current Research

Despite significant progress in understanding how fertilization regulates the rhizosphere microbiome, multiple challenges persist, hindering its translation into practical agricultural applications [111,112]. A core challenge lies in unraveling the complex causality. Fertilization practices can directly alter soil physicochemical properties (e.g., pH, nutrient availability), thereby filtering the microbial community [113]. Alternatively, they can indirectly drive microbial succession by modulating plant root exudates (e.g., the composition and quantity of sugars, organic acids) [114,115,116]. The intertwining of these direct and indirect effects makes it difficult to precisely pinpoint the dominant mechanisms within the “fertilization-plant-microbiome” triad [117]. For instance, after organic fertilizer application, is the observed increase in microbial diversity primarily due to introduced exogenous microbes or the promoted growth of indigenous microbes via improved soil conditions? Current research often relies on correlative analyses, lacking validation through causal experiments (e.g., grafting experiments or gnotobiotic systems), leading to ambiguous mechanistic explanations [118].

A significant gap between laboratory findings and field application impedes the practical use of microbial inoculants. Under controlled conditions (e.g., pot experiments), inoculating with probiotics (e.g., *Bacillus* or *Pseudomonas*) often significantly promotes crop growth and suppresses diseases. However, upon introduction to the field, these microbes face complex environmental stresses (e.g., temperature fluctuations, water stress, competition from indigenous microbiota), leading to a substantial decrease in their colonization efficiency and functional stability [119,120]. The heterogeneity of field soils (e.g., spatially distributed nutrient patches) further exacerbates the uncertainty of inoculant survival [121]. Moreover, current technologies for the production, storage, and application of microbial formulations are often immature, making it difficult to ensure the alignment of inoculant viability with field requirements [122].

The interplay among plant genotype, microbiome, and fertilization has not been systematically elucidated. Different crop varieties, or even different lines of the same variety, have genetic differences in their root exudate profiles, which recruit specific microbial taxa [123,124]. Fertilization strategies may amplify or weaken this genotype-dependent microbial assembly [125]. For example, would high phosphorus fertilization disrupt the interaction where low-phosphorus-tolerant rice varieties enrich phosphate-solubilizing bacteria by secreting specific organic acids? Current research predominantly focuses on single-factor effects, lacking multi-factorial experimental designs (e.g., Genotype × Fertilization × Microbiome), which limits the development of precise management strategies.

### 5.2. Future Research Directions

To overcome the challenges outlined above, future research needs to innovate methodological frameworks and maintain a strong application-oriented focus. The primary directions are as follows. A paramount direction is to deepen the integrated application of multi-omics technologies. Systematically elucidating the molecular basis of the “plant-microbe” dialog under fertilization can be achieved by leveraging metagenomics to reveal the microbial functional gene pool, meta-transcriptomics to dynamically track active metabolic pathways, and metabolomics to decipher the interaction networks between root exudates and microbial metabolites. For example, integrating spatiotemporal transcriptomics with metabolic flux analysis can precisely identify how plant signaling molecules (e.g., flavonoids), altered by fertilization, regulate the expression of microbial phosphate transformation genes, thereby uncovering the mechanisms behind nutrient-use efficiency.

The development of intelligent fertilization strategies is key to achieving precision agriculture [126]. Utilizing rapid soil microbiome detection technologies (e.g., portable sequencing devices or biosensors) to monitor field microbial community structure and the abundance of key functional microbes in real time will allow for the construction of predictive models linking fertilization, microbiome, and yield. Based on these models, fertilization schemes can be optimized dynamically; for instance, automatically recommending probiotic inoculation when pathogen abundance increases or adjusting nitrogen fertilizer application when diazotrophs are deficient [127]. Coupling these approaches with the Internet of Things (IoT) and artificial intelligence (AI) to establish a “soil-microbiome-climate” big data platform can enable dynamic, on-demand fertilization management.

Cultivating crop varieties that promote a beneficial rhizosphere microbiome represents a revolutionary direction [128,129]. This can be achieved by employing genome-wide association studies (*GWAS*) to identify host gene loci controlling root exudate synthesis, thereby screening germplasm resources capable of recruiting beneficial microbes (e.g., N2-fixing bacteria, disease-suppressing bacteria). Gene editing technologies (e.g., *CRISPR*) can be used to precisely modify root exudation traits, breeding “microbe-recruiting” crops [130,131]. For instance, optimizing rice genes responsible for organic acid secretion could enhance their ability to recruit phosphate-solubilizing microorganisms, reducing dependence on phosphorus fertilizers. This approach would empower the plant itself to act as an “engineer” of its microbiome, propelling agriculture towards a fully biological-driven paradigm (Table 4).

## Figures and Tables

**Table 1:** Core functions of the rhizosphere microbiome and their mechanisms of action.

Core Function Category	Specific Action/Process	Key Actors (Microbial Taxa/Factors)	Benefit to Plant/Soil	References
Nutrient Mobilization & Cycling	Drives biogeochemical cycles of key elements (e.g., N_2_ fixation, nitrification/denitrification, P solubilization, organic matter decomposition).	N_2_-fixing bacteria/archaea, nitrifying/denitrifying bacteria, P-solubilizing bacteria/fungi, Arbuscular Mycorrhizal Fungi (AMF).	Enhances soil nutrient availability and crop nutrient use efficiency.	[11,31,32,33]
Phytohormone Modulation	Synthesizes and modulates phytohormones (e.g., auxin/IAA); produces growth-promoting substances like siderophores.	Plant Growth-Promoting Rhizobacteria (PGPR, e.g., Pseudomonas, Bacillus).	Directly stimulates root development and growth, enhancing nutrient uptake.	[34,35]
Induced Systemic Resistance (ISR)	Enhances plant defense against pathogens by inducing systemic resistance.	Specific beneficial bacterial consortia/core microbial species.	Improves overall plant health, reducing disease incidence and reliance on chemical pesticides.	[1,24,36]
Rhizosphere Structure Modulation	Promotes soil aggregate formation and stabilizes soil structure via secretion of extracellular polymeric substances (EPS) and hyphal networks.	Plant roots (EPS secretion), mycorrhizal fungi, expolysaccharide-producing bacteria.	Improves rhizosphere aeration, water infiltration and retention, creating a favorable physical microenvironment.	[37]

**Table 2:** Differences among various fertilization strategies.

Fertilization Strategy	Impact on Microbial Diversity	Impact on Community Structure	Impact on Abundance of Key Functional Microbes	References
Mineral Fertilizer	Often reduces microbial diversity, e.g., long-term application decreases bacterial and fungal diversity.	Alters community structure, e.g., increases *Bacteroidetes* and decreases *Acidobacteria*; causes soil acidification, increasing environmental filtering pressure.	Reduces the abundance of beneficial microbes such as plant growth-promoting rhizobacteria (PGPR) and diazotrophs (e.g., *Burkholderia*).	[38,39,40,42]
Organic Fertilizer	Significantly increases microbial diversity, e.g., enhances the Shannon index and richness of bacteria and fungi.	Improves community structure by increasing beneficial phyla (e.g., *Proteobacteria*, *Actinobacteria*) and enhancing microbial network complexity.	Increases the abundance of key functional microbes like PGPR, phosphate-solubilizing bacteria, and diazotrophs, promoting nutrient cycling.	[87,88,89]
Bio-organic Fertilizer	Further increases microbial diversity, outperforming organic fertilizer alone, e.g., raises bacterial and fungal diversity indices.	Introduces beneficial microbes (e.g., *Bacillus*, *Pseudomonas*), shifts community structure, and increases co-occurrence network complexity and stability.	Significantly enriches specific beneficial microbes (e.g., *Trichoderma*, *Pseudomonas*) and suppresses pathogens.	[74,77,90]
Biochar	Increases microbial diversity, especially at high application rates, boosting bacterial and fungal diversity.	Affects phylum-level structure, e.g., increases *Proteobacteria*; alters microbial interaction networks.	Enhances the abundance of certain beneficial microbes (e.g., *Burkholderia*, *Gemmatimonas*), improving nutrient utilization.	[63,91,92]
Green Manure	Increases microbial diversity, e.g., increasing bacterial diversity in intercropping systems.	Improves microbial community structure by enriching beneficial bacteria (e.g., *Rhizobium*) and promotes soil health.	Increases the abundance of key functional microbes like diazotrophs and phosphate-solubilizing bacteria, enhancing ecosystem functions.	[57,61,93]
Microbial Inoculant	Increases or alters microbial diversity, e.g., inoculation with PGPR or AMF enhances bacterial and fungal diversity.	Significantly modifies community structure by introducing exogenous beneficial microbes, strengthening microbial interactions and network stability.	Directly increases the abundance of beneficial microbes (e.g., *Bacillus*, *Pseudomonas*, AMF), enhancing plant stress resistance.	[14,94,95]

Note: Biochar is essentially a soil amendment rather than a nutrient fertilizer. It is listed as an independent fertilization strategy here according to its common single or combined application mode in agricultural field trials.

**Table 3:** Pathways of fertilization-mediated feedback effects on crop growth via the rhizosphere microbiome.

Feedback Type	Typical Fertilization Scenario	Impact on Rhizosphere Microbiome	Final Effect on Crop Growth	Core Mechanism	References
Positive Feedback	Application of a compound microbial inoculant containing N_2_-fixing rhizobia and endophytes in a peanut-rice rotation system.	Significantly increases nodule nitrogenase activity (*nifH* gene expression +200%) and promotes plant uptake of N and P.	Peanut grain yield reaches 1168 kg ha^−1^, significantly higher than chemical fertilizer alone.	Direct enhancement of biological N fixation and nutrient mobilization via introduced functional microbes.	[107]
	Application of bio-organic fertilizer amended with *Trichoderma* in continuous cucumber cropping soil.	Enriches beneficial bacteria (e.g., *Sphingomonas*) and enhances activities of soil phosphatase and urease.	Improves P use efficiency, increases cucumber biomass by 20.39%, and reduces Fusarium wilt incidence.	Enriches indigenous beneficial flora and activates soil enzyme activity via probiotics, synergistically promoting growth and disease resistance.	[67]
Negative Feedback	Long-term excessive application of chemical N fertilizer.	Causes soil acidification, suppresses the abundance of AMF and diazotrophs, and reduces microbial network complexity.	Reduces crop nutrient acquisition and aggravates continuous cropping obstacles.	Soil acidification and disruption of biotic interaction networks lead to loss of key beneficial microbial functions.	[108]
	Sole application of chemical fertilizer in a continuous tomato cropping system.	Reduces rhizosphere microbial diversity, decreases abundance of antagonistic bacteria (e.g., *Pseudomonas*), and increases the relative abundance of pathogens (e.g., *Fusarium*).	Ultimately intensifies the occurrence of bacterial wilt.	Loss of microbial diversity and dysbiosis weaken the natural disease-suppressive barrier.	[109]
	Imbalanced fertilization (e.g., skewed nutrient ratios).	Disrupts microbial interaction networks and decreases functional redundancy.	Weakens soil’s buffering capacity against biotic stresses, leading to crop yield reduction and quality deterioration.	Impairment of ecological network stability and functional buffering capacity.	[110]

**Table 4:** Key challenges and corresponding future directions in rhizosphere microbiome research.

Key Challenge	Specific Issues/Difficulties	Future Research Direction	Key Technologies/Methods	Expected Outcome/Solution Path
Complexity of Causal Relationships Negative Feedback	The intertwined direct (altering soil properties) and indirect (modulating root exudates) effects of fertilization make it difficult to parse the dominant mechanisms in the “fertilization-plant-microbiome” triad. Current studies often rely on correlative analyses, lacking causal experimental validation.	Deepening Integrated Multi-Omics Applications	Metagenomics, metatranscriptomics, metabolomics, spatiotemporal transcriptomics, metabolic flux analysis.	Systematically elucidate the molecular basis of the “plant-microbe” dialog under fertilization, and precisely identify the mechanisms by which signaling molecules (e.g., flavonoids) regulate microbial functional gene expression.
The Lab-to-Field Gap	Microbial inoculants show significant effects in controlled experiments but suffer from drastically reduced colonization efficiency and functional stability in complex field environments. Soil heterogeneity, environmental stress, and competition from indigenous microbiota are major obstacles. Production, storage, and application technologies for formulations are immature.	Developing Smart Fertilization Strategies	Rapid soil microbiome detection (portable sequencing, biosensors), Internet of Things (IoT), Artificial Intelligence (AI), predictive modeling.	Build a “soil-microbiome-climate” big data platform and predictive models to achieve on-demand, dynamic, and precise fertilization based on real-time microbial community data (e.g., auto-recommending inoculants or adjusting fertilizer rates).
Poorly Understood Plant Genotype–Microbiome–Fertilization Interactions	Plant genotype regulates specific microbial assembly via root exudates. Fertilization may amplify or weaken this genotype-dependency. Current research lacks multi-factorial (Genotype × Fertilization × Microbiome) experimental designs, limiting the development of precise management strategies.	Breeding Crops for a Beneficial Rhizosphere Microbiome	Genome-wide association studies (GWAS), gene editing (e.g., *CRISPR*), germplasm screening, synthetic microbial community (SynCom) testing.	Identify host genes controlling root exudate synthesis, use gene editing to precisely modify root exudation traits, and breed “microbe-recruiting” crop varieties capable of actively recruiting beneficial microbes (e.g., *PSB*, diazotrophs), empowering the plant to act as an “engineer” of its own microbiome.

## Data Availability

The data that support the findings of this study are available from the corresponding author upon reasonable request.
